# Morphological and molecular description of *Ixodes woyliei* n. sp. (Ixodidae) with consideration for co-extinction with its critically endangered marsupial host

**DOI:** 10.1186/s13071-017-1997-8

**Published:** 2017-02-07

**Authors:** Amanda Ash, Aileen Elliot, Stephanie Godfrey, Halina Burmej, Mohammad Yazid Abdad, Amy Northover, Adrian Wayne, Keith Morris, Peta Clode, Alan Lymbery, R. C. Andrew Thompson

**Affiliations:** 1 School of Veterinary and Life Sciences, Murdoch University, 90 South St, Murdoch, WA 6150 Australia; 2 Environmental and Emerging Diseases Unit, Papua New Guinea Institute of Medical Research, Goroka, Papua New Guinea; 3 Science and Conservation Division, Western Australian Department of Parks and Wildlife, Manjimup, WA 6258 Australia; 4 Science and Conservation Division, Western Australian Department of Parks and Wildlife, Woodvale, WA 6946 Australia; 5 Centre for Microscopy, Characterisation and Analysis, The University of Western Australia, Stirling Highway, Perth, WA 6009 Australia

**Keywords:** *Ixodes woyliei* n. sp, Co-extinction, Ectoparasites, *Bettongia penicillata*, Wildlife

## Abstract

**Background:**

Taxonomic identification of ticks obtained during a longitudinal survey of the critically endangered marsupial, *Bettongia penicillata* Gray, 1837 (woylie, brush-tailed bettong) revealed a new species of *Ixodes* Latrielle, 1795. Here we provide morphological data for the female and nymphal life stages of this novel species (*Ixodes woyliei* n. sp.), in combination with molecular characterisation using the mitochondrial cytochrome *c* oxidase subunit 1 gene (*cox*1). In addition, molecular characterisation was conducted on several described *Ixodes* species and used to provide phylogenetic context.

**Results:**

*Ixodes* spp. ticks were collected from the two remaining indigenous *B. penicillata* populations in south-western Australia. Of 624 individual *B. penicillata* sampled, 290 (47%) were host to ticks of the genus *Ixodes*; specifically *I. woyliei* n. sp., *I. australiensis* Neumann, 1904, *I. myrmecobii* Roberts, 1962, *I. tasmani* Neumann, 1899 and *I. fecialis* Warburton & Nuttall, 1909. Of these, 123 (42%) were host to the newly described *I. woyliei* n. sp. In addition, 268 individuals from sympatric marsupial species (166 *Trichosurus vulpecula hypoleucus* Wagner, 1855 (brushtail possum), 89 *Dasyurus geoffroii* Gould, 1841 (Western quoll) and 13 *Isoodon obesulus fusciventer* Gray, 1841 (southern brown bandicoot)) were sampled for ectoparasites and of these, *I. woyliei* n. sp. was only found on two *I. o. fusciventer.*

**Conclusions:**

Morphological and molecular data have confirmed the first new Australian *Ixodes* tick species described in over 50 years, *Ixodes woyliei* n. sp. Based on the long-term data collected, it appears this tick has a strong predilection for *B. penicillata*, with 42% of *Ixodes* infections on this host identified as *I. woyliei* n. sp*.* The implications for this host-parasite relationship are unclear but there may be potential for a future co-extinction event. In addition, new molecular data have been generated for collected specimens of *I. australiensis, I. tasmani* and museum specimens of *I. victoriensis* Nuttall, 1916, which for the first time provides molecular support for the subgenus *Endopalpiger* Schulze, 1935 as initially defined. These genetic data provide essential information for future studies relying on genotyping for species identification or for those tackling the phylogenetic relationships of Australian *Ixodes* species.

## Background

Tick species within the genus *Ixodes* Latreille, 1795 (Ixodidae) have a worldwide distribution and can be found on a variety of hosts including mammals, birds, and occasionally reptiles. Species of *Ixodes* that generally command the greatest importance are those that are potential pathogens either directly as a cause of anaemia/blood loss or paralysis, and/or indirectly as a vector for disease. The role of ticks as vectors for disease has been the dominant area of research in recent times and many pathogenic protozoan, bacterial and viral agents have been isolated from a range of *Ixodes* species [1–4]. While the vectorial significance of ticks is well established, there are still many unknowns with respect to the genetic diversity, species delimitation and host distribution of ticks, particularly in wildlife. Recent studies using both morphological and molecular tools have confirmed new tick species from bats, wild boar, peccary, opossums, lizards and foxes [5–9], emphasizing the potential for new discoveries in wildlife populations.

Australian fauna are host to a large number of tick species, many of which are endemic to the Australasian region. Within the Australian lineage of the genus *Ixodes* there are currently 22 known species, with the latest species being described in 1962 [10–12]. The Australian tick descriptions provided by Roberts [12] remain the cornerstone of taxonomic identification even today, but due to the lack of sufficient specimens available at that time it is very likely that there are undiscovered tick species and unknown host associations.

The ability to discover either new tick species or new host records is greatly enhanced when longitudinal research is conducted on a large number of hosts. Such research has recently been undertaken on the critically endangered marsupial, *Bettongia penicillata* Gray, 1837 (Potoroidae) (woylie, brush-tailed bettong), of which indigenous populations are now restricted to the south-western corner of Australia. Investigations into rapid population declines experienced by this marsupial commenced in 2006 and are still ongoing [13–15]. Parasite infections were considered a possible contributing factor to these population declines and hence during this ten year period comprehensive ecto- and endoparasite data have been accumulated. Large numbers of ticks were also collected from many individual hosts from a range of other species throughout a range of seasonal conditions over this period.

Taxonomic identification of ticks obtained from trapped *B. penicillata* revealed a new *Ixodes* species first detected in 2007 [15] and again following the longitudinal surveillance of several populations from 2014 to 2016 [16]. Morphologically, this unidentified tick was similar to *Ixodes* species within the subgenus *Endopalpiger* Schulze, 1935 as described by Roberts [12], of which four species had been described, *Ixodes victoriensis* Nuttall, 1916, *I. australiensis* Neumann, 1904, *I. tasmani* Neumann, 1899 and *I. hydromyidis* Swan, 1931. Indeed, initial identification of this tick using dichotomous keys developed by Roberts [12] indicated this tick was *I. victoriensis*, a tick that to date has only been found on wombats and potoroos in Victoria and Tasmania [17]. However, this identification was considered to be incorrect due to significant differences observed between the two species particularly the shape of the scutum, palpal article 1, and spurs on the coxae. These same morphological differences were also recently highlighted by Weaver [17], who examined four specimens labelled as *I. victoriensis* kept at the Australian National Insect Collection (ANIC). These specimens had been collected from *B. penicillata* in western Australia, but due to geographical location and subsequent redescription, Weaver [17] considered these to be a misidentification.

Here, we provide morphological data for the female and nymphal life stages of this novel *Ixodes* species, in combination with molecular characterisation using the mitochondrial cytochrome *c* oxidase subunit 1 gene (*cox*1). Due to minimal genetic information available for Australian *Ixodes* species, molecular characterisation was also conducted on several described species and used to provide phylogenetic context. The conservation implications for this novel *Ixodes* species, which appears to have a predilection for an endangered marsupial as a host, are also considered.

## Methods

### Tick collection and morphological identification

**Fig. 1:**
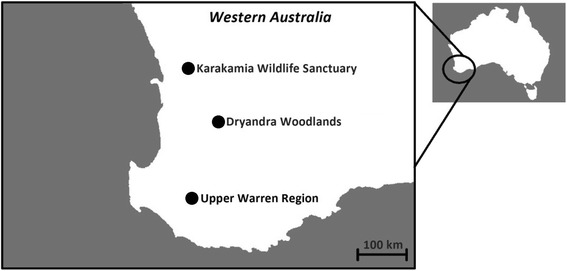
Locations of *Bettongia penicillata* populations where samples were obtained

*Ixodes* spp. ticks were collected from 2006 to 2016 from the two remaining indigenous *B. penicillata* populations in south-western Australia (Dryandra Woodland and the Upper Warren Region), and a translocated population near Perth (Karakamia Wildlife Sanctuary) (Fig. 1). Ticks were also collected from sympatric species in Dryandra Woodland and the Upper Warren, including *Trichosurus vulpecula hypoleucus* Wagner, 1855 (koomal, brush tail possum), *Dasyurus geoffroii* Gould, 1841 (chuditch, western quoll) and *Isoodon obesulus fusciventer* Gray, 1841 (quenda, southern brown bandicoot). The tick collection held at Murdoch University Parasitology Section Museum was also scrutinized for unidentified *Ixodes* spp. that could potentially belong to the new tick species. Specimens of *I. victoriensis* currently held at ANIC were obtained for morphological comparisons. All tick specimens were preserved in 70% ethanol and later identified using keys developed by Roberts [12].

Morphometric data were based on 12 adult female specimens (four unfed, seven partially fed and one engorged) and 14 nymph specimens (nine unfed, four fed and one engorged). To date male specimens of the new species have not been collected. All except two nymphs were collected from *B. penicillata* located in the Dryandra Woodland and the Upper Warren region and one animal housed at Perth Zoo. The two exceptions were museum specimens collected from *Macrotis lagotis* Reid, 1837 (greater bilby) housed at Kanyana Wildlife Rehabilitation Centre Perth W.A. Measurements (in millimetres unless indicated otherwise) were taken from specimens temporarily mounted on slides. Selected specimens were cleared in lactophenol for photographing with line drawings done to scale from these images.

Six specimens were also prepared for observation by scanning electron microscopy (SEM). Samples were fully dehydrated in 100% anhydrous ethanol and critical point dried, before being mounted on stubs with carbon tape and coated with ~20 nm gold. All imaging was done at 10–15 kV on a Zeiss field emission SEM.

### DNA extraction

Adult female and nymph specimens identified as the new *Ixodes* species were chosen for molecular characterisation, along with specimens identified as *I. australiensis*, *I. tasmani*, *I. victoriensis*, *I. myrmecobii* and *I. fecialis*. Ethanol-preserved ticks were rehydrated in a series of decreasing ethanol concentrations. Specifically specimens were successively placed for 1 h each in 50%, 30% and 10% ethanol with the final hour in 100% dH_2_0. Following rehydration, specimens were roughly dissected with fresh disposable scalpel blades before being frozen in liquid nitrogen and ground as finely as possible. All specimens were then digested with proteinase K overnight at 56 °C before DNA extraction using the Maxwell® 16 instrument (Promega, Madison, USA) or with the Qiagen DNeasy blood and tissue kit (Hilden, Germany).

### PCR amplification and sequencing

All tick specimens were amplified by PCR at the *cox*1 gene with minor modifications from a previously described protocol [18]. PCR reactions were performed in 25 μl volumes consisting of 1–2 μl of extracted DNA, 2.0 mM MgCl_2_, 1× reaction buffer (20 mM Tris-HCL, pH 8.5 at 25 °C, 50 mM KCl), 200 μM of each dNTP, 0.4 μM of each primer, and 1 unit of Taq DNA polymerase (Fisher Biotec, Perth, Australia). Amplification conditions for *cox*1 involved a denaturing step of 95 °C for 5 min, 40 cycles of 95 °C for 45 s, 50–51 °C for 60 s and 72 °C for 60 s, followed by a final extension of 72 °C for 5 min. PCR products were purified using an Agencourt AMPure XP system (Beckman Coulter Inc., Brea, USA) and sequence reactions were performed using the Big Dye Terminator Version 3.1 cycle sequencing kit (Applied Biosystems, Foster City, USA) according to the manufacturer’s instructions. Reactions were electrophoresed on an ABI 3730 96 capillary machine.

### Phylogenetic analyses

Resultant sequences were compared with available published sequences on GenBank using the basic alignment search tool (BLAST) with further analysis of sequence alignments conducted in Sequencher® V5.2.4 (Gene Codes Corporation, Ann Arbor, USA). Additional sequences retrieved from GenBank representing *I. holocyclus* Neumann, 1899 (AB075955, HM545841), *I. cornuatus* Roberts, 1960 (KM821527, HM545846), *I. hirsti* Hassall, 1931 (KM821524), *I. fecialis*, (FJ571509), *I. uriae* White, 1852 (NC006078) and *Rhipicephalus sanguineus* Latrielle, 1806 (JX416308) were included in the phylogenetic analyses conducted in MEGA7 [19]. Phylogenetic trees were inferred with the neighbour-joining method, with a bootstrapping of 1,000 replicates and evolutionary distances calculated using the Kimura 2-parameter method [20, 21]. In addition analyses were conducted using the maximum likelihood and maximum parsimony methods [22].

## Results

### Detection of *Ixodes* spp.

Of 624 individual *B. penicillata* sampled between 2006 and 2016, 290 (47%) were host to ticks of the genus *Ixodes*. Of these, 123 (42%) were host to the new species described below (Table 1). In addition, 268 individuals of sympatric species (166 *T. v. hypoleucus,* 89 *D. geoffroii* and 13 *I. o. fusciventer*) were sampled for ectoparasites and of these, the new species was found on two *I. o. fusciventer* (Table 1). Within the museum collection held at Murdoch University, additional specimens of the new species were identified from five individual *B. penicillata* and one *M. lagotis*. Further information regarding the hosts of these museum specimens was not available.

**Table 1:** The species composition of *Ixodes* spp. detected from *B. penicillata* and sympatric species during trapping sessions conducted from 2006 through to 2016. The proportions of each *Ixodes* sp. among infected individuals are given as a percentage in parentheses

	*Bettongia penicillata* (Woylie)	*Trichosurus vulpecula hypoleucus* (Brushtail possum)	*Dasyurus geoffroii* (Western quoll)	*Isoodon obesulus fusciventer* (Southern brown bandicoot)
Individuals sampled	624	166	89	13
Individuals host to an *Ixodes* sp.	290 (46.5%)	63 (38%)	22 (24.7%)	5 (38.5%)
*Ixodes woyliei* n. sp.	123 (42.4%)	0	0	2 (40%)
*Ixodes australiensis*	153 (52.7%)	1 (1.5%)	13 (59%)	2 (40%)
*Ixodes myrmecobii*	52 (17.9%)	3 (4.7%)	0	1 (20%)
*Ixodes tasmani*	4 (1.3%)	54 (85.7%)	7 (31.8%)	0
*Ixodes fecialis*	1 (0.3%)	0	9 (40.1%)	0

**Family Ixodidae Dugés, 1834**

**Genus** ***Ixodes*** **Latreille, 1795**

### *Ixodes woyliei* n. sp

***Type-host***: *Bettongia penicillata* Gray, 1837 (Potoroidae) (woylie, brush-tailed bettong).

***Other hosts***: *Isoodon obesulus fusciventer* Shaw, 1797 (Peramelidae) (quenda, southern brown bandicoot) and *Macrotis lagotis* Reid, 1837 (Peramelidae) (greater bilby).

***Type-locality***: Dryandra Woodland, Western Australia, Australia (32°47'S, 116°58'E).

***Other localities***: Karakamia Wildlife Sanctuary, Western Australia, Australia (31°48'S, 116°15'E) and the Upper Warren Region, Western Australia, Australia (34°21'41"S, 116°18'22"E).

***Type-specimens***: Holotype: female ex *B. penicillata*, Dryandra Woodland, Western Australia, Australia (32°47'S 116°58'E), December 2015, deposited at the West Australian Museum (WAM T142602). Paratypes*:* Total 25, 11 females and 14 nymphs ex *B. penicillata.* Eight females (P1–P8) collected from Dryandra Woodland, Western Australia, Australia (32°47'S, 116°58'E), June 2016 (P1, P2, P7, P8), September 2015 (P3, P4), February 2015 (P5, P6); 2 females (P9, P10) collected from Karakamia Wildlife Sanctuary, Western Australia, Australia (31°48'S, 116°15'E) July 2006; and one female (P11) collected from the Upper Warren Region Western Australia, Australia (34°21'41"S, 116°18'22"E). Seven nymphs (P12–P18) collected from Upper Warren Region, Western Australia, Australia (34°21'41"S, 116°18'22"E) September 2014 and December 2014, 4 nymphs (P19–P22) collected from Dryandra Woodland, Western Australia, Australia (32°47'S, 116°58'E), September 1994 and October 1993, one (P23) collected from Perth Zoo and two (P23–25) collected from Kanyana Wildlife Rehabilitation Centre, Perth. Seven paratype specimens (P1, P2, P7, P8, P15–P17) have been deposited at the Australian National Insect Collection (ANIC 48-006275 – ANIC 48-006277) and five paratype specimens (P9, P10, P12–P14) have been deposited at the Western Australian Museum (WAM T142603-WAM T142604).

***Representative DNA sequences***: Mitochondrial cytochrome *c* oxidase subunit 1 (*cox*1): GenBank accession numbers KX673875–KX673881.

***ZooBank registration***: Details of *Ixodes woyliei* n. sp. have been submitted to ZooBank and the Life Science Identifier (LSID) for the article is urn:lsid:zoobank.org:pub:1DB97319-FF74-4380-9D49-988BA3467342 and LSID for the new name is urn:lsid:zoobank.org:act:7FC91916-ACBC-4832-8788-19CCBB8EF7B1.

***Etymology***: The species name *Ixodes woyliei* refers to the common name of the host *B. penicillata* (commonly known as the woylie) for which this tick appears to have a high predilection. Woylie is the Aboriginal name given by the Noongar people who live in the south-west corner of Western Australia [23].

### Description

***General***. Golden brown medium-sized ticks with greatly enlarged palpal article 1, over crowded hypostome dentition mainly 6/6 and 5/5, coxae all armed with strong pointed spurs, and anal groove open posteriorly.

**Fig. 2:**
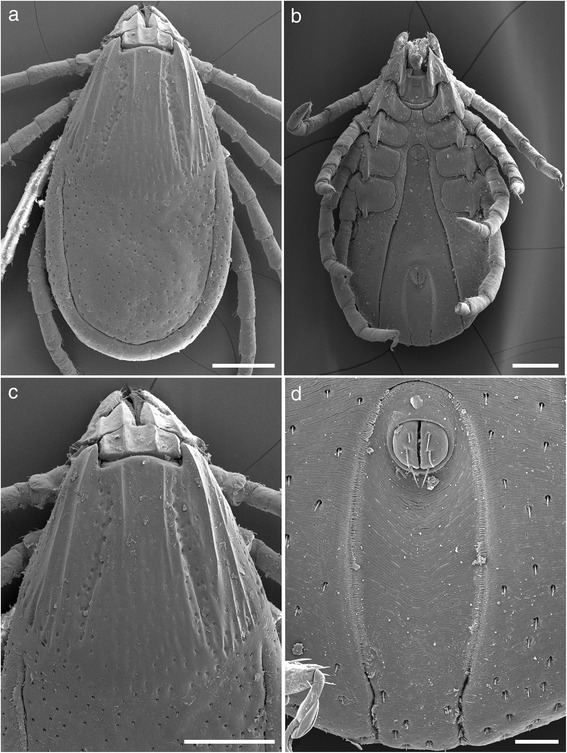
Scanning electron micrographs of *Ixodes woyliei* n. sp. Female. **a** Idiosoma, unengorged specimen, dorsal view. **b** Idiosoma, unengorged specimen, ventral view. **c** Scutum, showing lateral carinae. **d** Anal groove. *Scale-bars*: **a**-**c**, 500 μm; **d**, 100 μm

***Female***. Idiosoma (Fig. 2): Unfed specimens oval and elongate, widest just posteriorly of spiracles. Body length measured dorsally from midway between scapular points to most posterior margin range from 2.4–3.3, width 1.4–2.2 (Fig. 2a). Partially fed specimens length range from 3.3–5.3, width 1.9–2.5, engorged specimens attaining length 11, width 6.5. Dorsal setae are short (<25 μm), lay within a uniform moderate covering of shallow punctations, marginal grooves are well defined. The scutum about as long as wide, widest point posterior to mid-length, anterolateral and posterolateral margins are mildly sinuous with posterior angle broadly rounded, lateral carinae present (Figs. 2c and 5c). Scutal length range from 1.2–1.5, width 1.2–1.6. Punctations are shallow and moderate in number, becoming coarser in cervical grooves, lateral rugae and along the posterior margin; scutal setae are minute (<8 μm). Cervical grooves well defined anteriorly becoming shallow posteriorly and extending to, or almost to the scutal margin. Scapulae are large and bluntly pointed. Ventral setae are longer than dorsal (<40 μm) with two rows of longer setae (>40 μm) around the lateral side of the spiracular plate. Genital aperture is level with the anterior margin of coxa III, but moving towards second intercoxal space on engorgement (Fig. 2b). Spiracular plates suboval, length 0.16–0.30, with approximately 3–4 rows of goblets, macular eccentric (Fig. 4b). Anal groove is horseshoe shaped, rounded anteriorly, curving gently and convergently posteriorly but becoming slightly divergent near body margin and remaining widely open (Fig. 2d). Both the internal and external margin of the anal groove epicutical surface possess several rows of inward facing spines, overlapping across the divide of the anal groove and running laterally for most of its length. This feature has not been mentioned previously but appears to be typical for all *Ixodes* species that we have been able to examine.

**Fig. 3:**
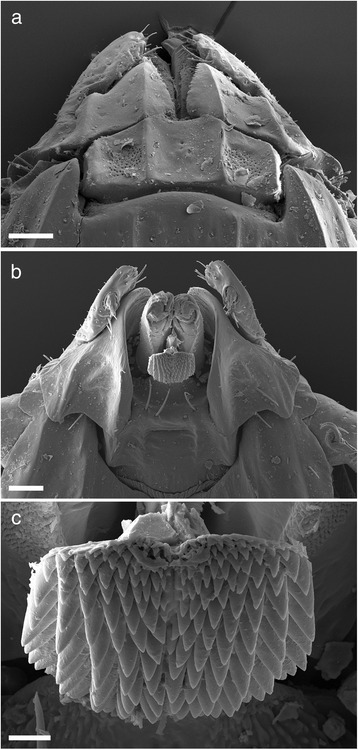
Scanning electron micrographs of *Ixodes woyliei* n. sp. Female. **a** Gnathosoma, dorsal view. **b** Gnathosoma, ventral view. **c** Hypostome. *Scale-bars*: **a**-**b**, 100 μm; **c**, 20 μm

Gnathosoma (Fig. 3) Basis dorsally, short, 0.36–0.60 in length by 0.40–0.55 in width. Length measurement taken from top of the palpi to the posterior margin of the cornua. Dorsal basis capituli subrectangular, with one median depression and a lateral depression on each side, the depressed areas being separated by carinae, posterior margin slightly undulating with small indistinct cornua (Figs. 3a and 5b). Porose areas large, suboval, lying in lateral depressions, widely separated by the median depression. Basis ventrally about as long as wide with small but distinct auriculae. Palps short and article 1 greatly enlarged, extending inwardly to partially ensheathe base of mouthparts, ventrally with a strong posterolateral salience. Articles 2 and 3 are without apparent suture, total length 0.29–0.35, width 0.07–0.15 (Figs. 3b and 5a). Hypostome length ranges from 0.17–0.33, width 0.15–0.17, spatulate, broad anteriorly, with sharply pointed large denticles, mainly 6/6 and 5/5. Dentition formula essentially 12/12 of small over crowded denticles at the corona, dropping in number but increasing in size to 6/6 and 5/5 by anterior third and reducing to 4/4, 3/3 with crenulations running down to the base (Fig. 3c).

**Fig. 4:**
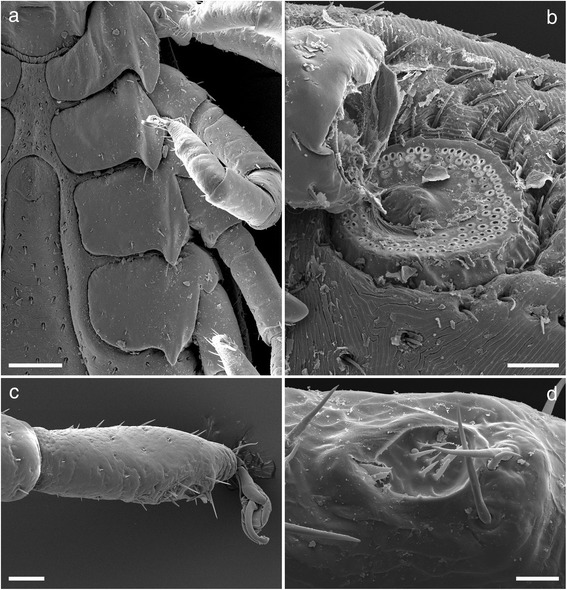
Scanning electron micrographs of *Ixodes woyliei* n. sp. Female. **a** Spurs on coxae. **b** Spiracular plate. **c** Tarsus I. **d** Haller’s organ. *Scale-bars*: **a**, 200 μm; **b**, **c**, 50 μm; **d**, 15 μm

**Fig. 5:**
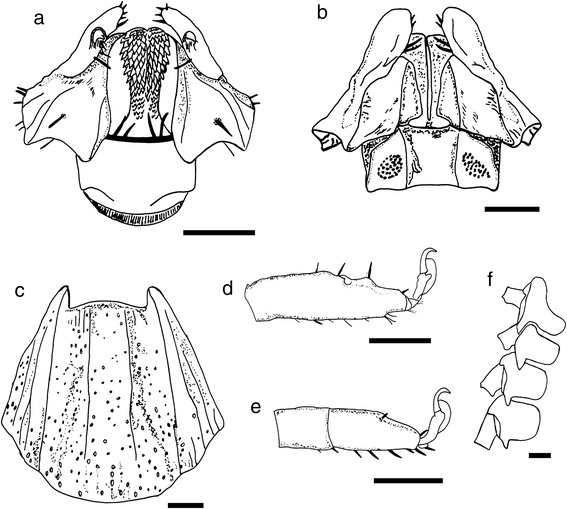
Line drawing of *Ixodes woyliei* n. sp. Female. **a** Capitulum, ventral view. **b** Capitulum, dorsal view. **c** Scutum. **d** Tarsis I. **e** Tarsis IV. **f** Coxae. *Scale-bars*: 200 μm

Legs (Fig. 4): Slender and moderate length. Coxa I transversely elongate with a strong pointed external spur. Coxae II, III, and IV somewhat square with progressively smaller pointed external spurs, all coxae with few setae, syncoxae absent (Figs. 4a and 5f). Length of tarsus I 0.3–0.5 with few long setae (<50 μm) and some small (<20 μm) (Figs. 4c and 5d, e). Haller’s organ, anterior pit suboval with seven sensilla arranged in a cluster in the centre, posterior capsule opening slightly above the pit and divided by a low ridge with up to five sensilla seen within (Fig. 4d). Length of tarsus IV is 0.4–0.5.

**Fig. 6:**
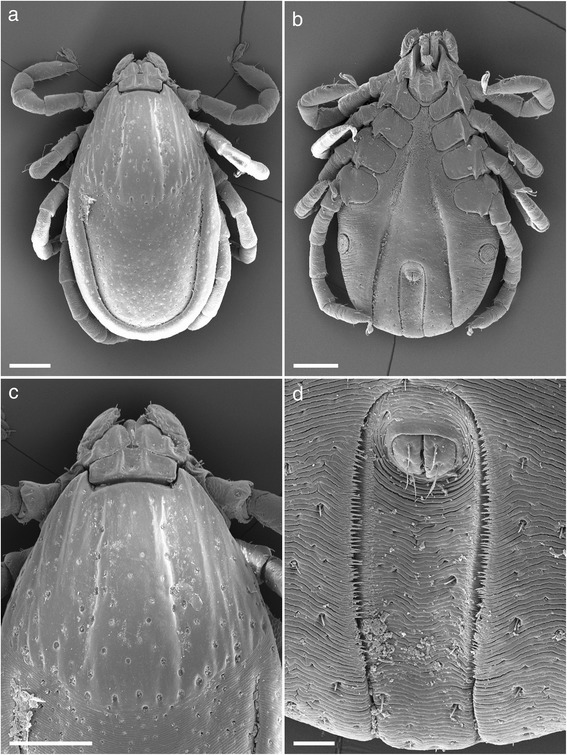
Scanning electron micrographs of *Ixodes woyliei* n. sp. Nymph. **a** Idiosoma, unengorged specimen, dorsal view. **b** Idiosoma, unengorged specimen, ventral view. **c** Scutum, showing lateral carinae. **d** Anal groove. *Scale-bars*: **a**-**c**, 200 μm; **d**, 40 μm

***Nymph***. Idiosoma (Fig. 6): Unfed specimens oval and elongate, widest about mid length between coxae III and IV, well-defined marginal grooves and minute dorsal setae (<10) within uniformly scattered punctations (Fig. 6a). Body length measured dorsally from midway between the scapular points to most posterior margin range from 1.3–1.6, width range 0.78–1.1. Fed specimen length range from 1.7–2.2, width 0.97–1.6, with engorged specimens attaining length 4.0, width 2.0. Scutum wider than long, with posterior angle broadly rounded, lateral carinae present (Figs. 6c and 9c). Scutal length ranges from 0.52–0.63, width 0.63–0.75. Punctations are shallow and moderate in number, becoming coarser in cervical grooves, lateral rugae and along the posterior margin; scutal setae are minute (<8 μm). Cervical grooves are well defined anteriorly becoming shallow posteriorly and extending to, or almost to the scutal margin. Scapulae are large and bluntly pointed. Ventral setae are slightly longer than dorsal mostly (<15 μm) with some longer setae (>20 μm) around the spiracular plate (Fig. 6b). Spiracles suboval, length 0.095–0.10, width 0.065–0.12 with *c*.3–4 rows of goblets covering the whole surface (Fig. 8b). Anal groove is horseshoe shaped, rounded anteriorly, curving gently and convergently posteriorly but becoming slightly divergent near body margin and remaining widely open (Fig. 6d). Both the internal and external margin of the anal groove epicutical possess several rows of inward facing spines as seen in the adult female.

**Fig. 7:**
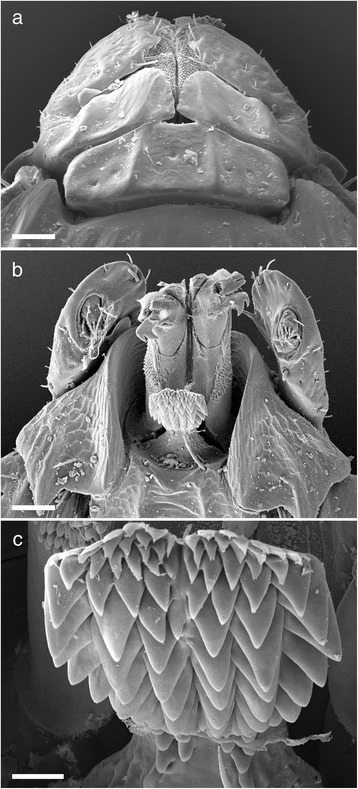
Scanning electron micrographs of *Ixodes woyliei* n. sp. Nymph. **a** Gnathosoma, dorsal view. **b** Gnathosoma, ventral view. **c** Hypostome. *Scale-bars*: **a**, **b** = 40 μm; **c**, 10 μm

Gnathosoma (Fig. 7): Basis dorsal length measurement taken from the top of the palpi to the posterior margin of the basis, length 0.19–0.26, width 0.22–0.26 in width. Dorsal basis capituli subrectangular, posterolateral angles roundly pointed (Figs. 7a ad 9b). Basis ventrally rounded posteriorly with small auriculae. Palps short and article 1 greatly enlarged, extending inwardly to partially ensheathe base of mouthparts, ventrally with a strong posterolateral salience. Articles 2 and 3 without apparent suture total length 0.15–0.17 by width 0.055–0.080 (Figs. 7b and 9a). Hypostome length ranges from 0.13–0.17, width 0.065–0.130, spatulate, broad anteriorly, with sharply pointed denticles, mainly 3/3. Dentition formula essentially 6/6 small denticles at corona then decreasing in number, but increasing in size to 4/4 followed by about 8 rows of 3/3 (Fig. 7c).

**Fig. 8:**
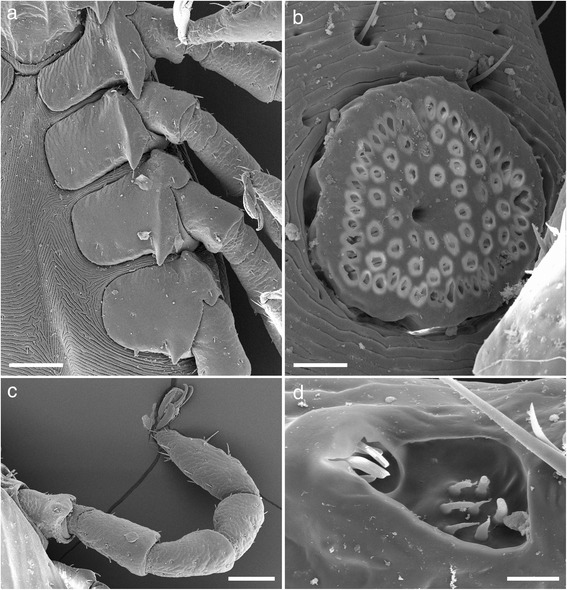
Scanning electron micrographs of *Ixodes woyliei*. Nymph, legs and spiracular plate. **a** Spurs on coxae. **b** Spiracular plate. **c** Tarsus I. **d** Haller’s organ. *Scale-bars*: **a**, **c**, 100 μm; **b**, 20 μm; **d**, 10 μm

**Fig. 9:**
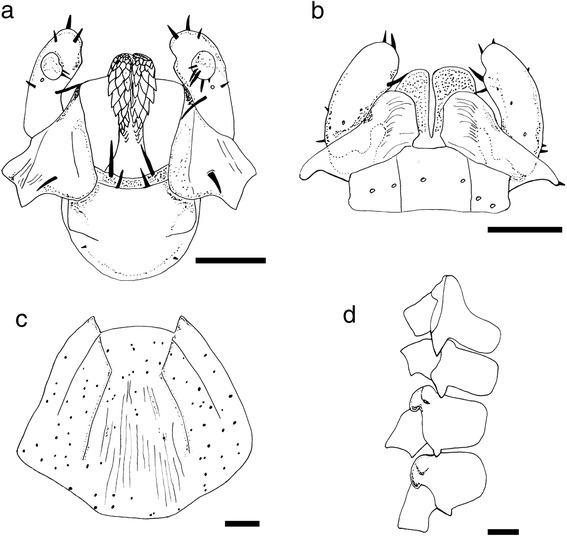
Line drawing of *Ixodes woyliei* n. sp. Nymph. **a** Capitulum ventral view. **b** Capitulum dorsal view. **c** Scutum. **d** Coxae. *Scale-bars*: 100 μm

Legs (Fig. 8): Slender and of moderate length. Coxa I transversely elongate with a strong pointed external spur. Coxae II, III, and IV somewhat square with progressively smaller pointed external spurs, all coxae with few setae, syncoxae absent (Figs. 8a and 9d). Length of tarsus I 0.22–0.30 with few long setae (<40 μm) and some minute (<10 μm) (Fig. 8c). Haller’s organ, anterior pit suboval with seven sensilla arranged in a cluster in the centre, posterior capsule opening slightly above the pit and divided by a low ridge with at least four sensilla visible (Fig. 8b). Length of tarsus IV is 0.20–0.30.

### Differential diagnosis

Morphologically, *I. woyliei* logically conforms to the subgenus *Endopalpiger* as described by Roberts [12] due to the enlarged palpal article 1 that extends inwardly to partially ensheathe the base of the mouthparts; common to all females within this subgenus. However it is also pertinent to consider the species of *Exopalpiger* Schulze, 1935 (*I. fecialis*, *I. vestitus* Neumann, 1908 and *I. antechini* Roberts, 1960) as more recently the species of these two subgenera have been amalgamated into *Exopalpiger* [24]. The species of these two subgenera however can be quite easily differentiated by the palpal article 1 which in *Exopalpiger* species is enlarged but does not extend inwardly, as seen in *I. woyliei* n. sp. and the other *Endopalpiger* species. Further morphological differences are outlined in Table 2. Differentiation of adult females from the other four *Endopalpiger* species can be achieved by the presence of an open anal groove, the large pointed spurs on each coxa, presence of syncoxae, the large number of denticles on the hypostome, and the shape of the scutum (Table 2). Specifically, the presence of an open anal groove, the lack of syncoxae and greater dentition differentiates *I. woyliei* n. sp. from *I. australiensis*, while the presence of spurs on the coxae (armed) differentiates *I. woyliei* n. sp. from *I. tasmani* and *I. hydromyidis*, both of which lack spurs. The most morphologically similar species to *I. woyliei* n. sp. is *I. victoriensis*; however these two species can be readily differentiated by dentition and the shape of the scutum, spurs on the coxae, and palpal article 1. *Ixodes woyliei* n. sp. has a remarkable and complex dentition with small overcrowded denticles at the corona (12/12), dropping in number but increasing in size to mainly a 6/6 and 5/5 dentition whereas *I. victoriensis* dentition is mostly 5/5, with rows of 4/4 at both anterior and posterior ends [17]. The shape of the scutum in *I. woyliei* n. sp. is longer than that of *I. victoriensis* (about as long as wide *vs* wider than long for *I. woyliei*), and appears more angular. The coxae of *I. woyliei* n. sp. are all armed with large, pointed spurs and lack syncoxae, while *I. victoriensis* coxae are armed with smaller spurs that are not as pointed and possess syncoxae. The enlarged palpal article 1 described for *I. woyliei* n. sp. has a posterolateral prominence making it more widely rectangular than that seen on *I. victoriensis*.

**Table 2:** Main morphological differences between adult female (F) and nymph (N) life stages of *Ixodes woyliei* and described species of *Exopalpiger* and *Endopalpiger*. Morphological differences between life stages are noted with (F) or (N); otherwise traits are the same for both

*Ixodes* spp.	Enlarged palpal article 1	Anal groove	Armed coxae	Syncoxae	Dentition	Cornua	Lateral carinae
*I. woyliei*	partially ensheathe mouthparts	open	yes	no	6/6 (F) 4/4 (N)	mild (F) no (N)	yes
*I. australiensis* ^a^	partially ensheathe mouthparts	closed (F) open (N)	yes	yes	4/4	yes (F) no (N)	no
*I. tasmani* ^a^	partially ensheathe mouthparts	open	no	yes	4/4 (F) 2/2 (N)	no	no
*I. hydromydis* ^a^	partially ensheathe mouthparts	open	no	yes	3/3 (F) 2/2 (N)	no	no
*I. victoriensis* ^b^	partially ensheathe mouthparts	open	yes	yes	5/5 (F) 4/4 (N)	yes (F) no (N)	yes
*I. fecialis* ^a^	do not ensheathe mouthparts	open	no	yes	2/2	no	yes
*I. vestitus* ^a^	do not ensheathe mouthparts	open	no	no	2/2	no	fine
*I. antechini* ^a^	do not ensheathe mouthparts	open	one minute on 1st coxa	yes	2/2	no	yes

^a^Description as per Roberts [1]

^b^Description as per Weaver [17]

Differential diagnosis of the nymphal stage can be largely achieved as for the adult female (Table 2). A minor exception involves *I. australiensis* whereby the nymph has an anal groove which remains open, but not as widely as seen in *I. woyliei* nymphs. However the shape of palpal article one, spurs and presence of syncoxae allow differentiation.

### Molecular characterisation

*Cox*1 gene sequences were obtained for 27 *Ixodes* spp. ticks; eight *I. woyliei* n. sp., eight *I. australiensis*, five *I. tasmani*, two *I. victoriensis*, two *I. fecialis* and two *I. myrmecobii* (Table 3). All available life-stages for each species were successfully amplified and sequenced. Sequence alignment of the ~800 bp product revealed single nucleotide polymorphisms (SNPs) ranging from zero within *I. victoriensis* to 32 within *I. woyliei*, although the small sample sizes for some species make this variation in SNPs difficult to interpret.

**Table 3:** Tick specimen information for all molecular data generated in this study

Sample ID	*Ixodes* spp.	Tick life stage	Host	GenBank accession numbers
T5	*I. woyliei*	Female	*Bettongia penicillata*	KX673875
T24	*I. woyliei*	Female	*Bettongia penicillata*	not obtained
T26	*I. woyliei*	Nymph	*Bettongia penicillata*	KX673876
T27	*I. woyliei*	Female	*Bettongia penicillata*	KX673877
T39	*I. woyliei*	Female	*Bettongia penicillata*	KX673878
T40	*I. woyliei*	Female	*Bettongia penicillata*	KX673879
T41	*I. woyliei*	Nymph	*Bettongia penicillata*	KX673880
T42	*I. woyliei*	Nymph	*Bettongia penicillata*	KX673881
T1	*I. australiensis*	Female	*Bettongia penicillata*	KX673858
T2	*I. australiensis*	Nymph	*Bettongia penicillata*	KX673859
T3	*I. australiensis*	Nymph	*Bettongia penicillata*	KX673860
T6	*I. australiensis*	Male	*Bettongia penicillata*	KX673861
T7	*I. australiensis*	Female	*Bettongia penicillata*	KX673862
T8	*I. australiensis*	Male	*Bettongia penicillata*	KX673863
T10	*I. australiensis*	Female	*Dasyurus geoffroii*	KX673864
T11	*I. australiensis*	Female	*Bettongia penicillata*	KX673865
T17	*I. tasmani*	Female	*Trichosurus vulpecula*	KX673866
T18	*I. tasmani*	Female	*Trichosurus vulpecula*	KX673867
T19	*I. tasmani*	Nymph	*Trichosurus vulpecula*	KX673868
T20	*I. tasmani*	Nymph	*Trichosurus vulpecula*	KX673869
T21	*I. tasmani*	Female	*Trichosurus vulpecula*	KX673870
T22	*I. fecialis*	Female	*Dasyurus geoffroii*	KX673871
T23	*I. fecialis*	Female	*Dasyurus geoffroii*	KX673872
T13	*I. myrmecobii*	Nymph	*Trichosurus vulpecula*	KX673882
T14	*I. myrmecobii*	Male	*Bettongia penicillata*	KX673883
T33	*I. victoriensis*	Larva	*Potorous longipes*	KX673873
T34	*I. victoriensis*	Nymph	*Potorous longipes*	KX673874

### Phylogenetic analyses

**Fig. 10:**
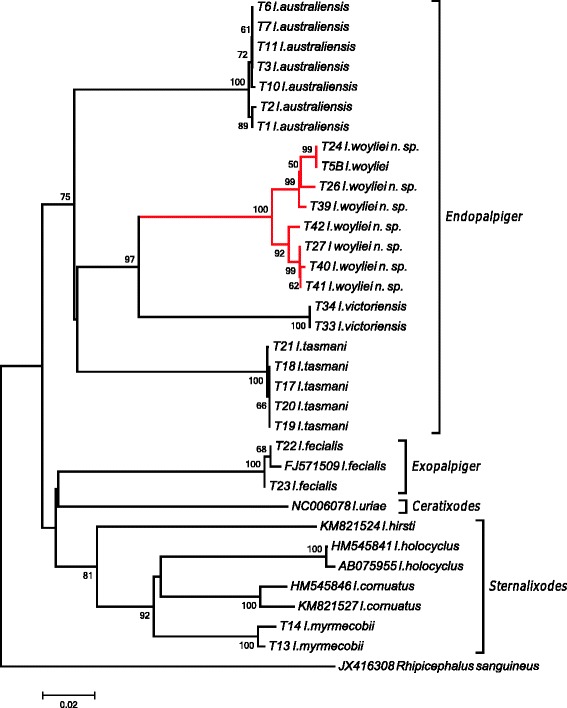
Phylogenetic relationships of isolates of *Ixodes woyliei* n. sp. with other Australasian *Ixodes* spp. as estimated using cytochrome *c* oxidase subunit 1 (*cox*1) gene sequences. Sequences with accession numbers were obtained from GenBank, all others were generated in this study. Evolutionary history was inferred using the neighbour-joining method supported with bootstrap test of 1,000 replicates (values > 50% shown). *Rhipicephalus sanguineus* is used as the outgroup

Phylogenetic analyses were conducted with all sequences obtained from this study, along with available published sequences for *I. holocyclus*, *I. cornuatus*, *I hirsti*, *I. uriae*, *I fecialis* and *Rhipicephalus sanguineus.* This allowed for four of the five subgenera within the Australasian *Ixodes* spp*.*, as described by Roberts [12], to be represented; namely *Endopalpiger*, *Exopalpiger*, *Sternalixodes* Schulze, 1935 and *Ceratixodes* Neumann, 1902. Trees constructed using neighbour-joining, maximum likelihood and maximum parsimony methods gave a similar topology, hence only the NJ tree is presented here (Fig. 10). The trees displayed consistency in placement of *I. woyliei* n. sp. as a sister species of *I. victoriensis* and in clustering with *I. australiensis* and *I. tasmani*; all *Endopalpiger* species. Similarly, the sequences generated from *I. myrmecobii* specimens consistently clustered with the other species representing the subgenus *Sternalixodes*, *I. holocyclus*, *I. cornuatus* and *I. hirsti*. Generated sequences for *I. fecialis*, representing the subgenus *Exopalpiger*, matched the published sequence (FJ571509) but due to lack of genetic material for other *Exopalpiger* species a grouping was not conclusive.

## Discussion

### Molecular confirmation of *I. woyliei* n. sp.

The molecular data generated for *I. woyliei* n. sp. conclusively supports the taxonomy, with *I. woyliei* positioned in a monophyletic group with the other *Endopalpiger* species for which genetic data were obtained, namely *I. australiensis*, *I. tasmani* and *I. victoriensis*. This also provides the first molecular support for the subgenus *Endopalpiger*. The close morphological relationship between *I. woyliei* n. sp. and *I. victoriensis* is also supported genetically, with the two positioning as sister species within this monophyletic grouping. Interestingly, the positioning of the one species of *Exopalpige*r genotyped, *I. fecialis*, does not support the monophyletic grouping of *Endopalpiger* and *Exopalpiger* as per Camaicas [24] but that of Roberts [12]. However, more genetic data are required to confirm or deny these taxonomic groupings and would require further research.

Genetically it appears that *I. woylie* n. sp. is a distinct species, but it is also necessary to consider the presence/absence of genetic exchangeability between groups to be confident of species status [25, 26]. *Ixodes woyliei*, *I. australiensis* and *I. tasmani* are sympatric, sharing the same geographical region, habitat and host; yet they remain genetically distinct. This would infer a lack of genetic exchange and therefore distinct species. This is not the case with *I. woyliei* n. sp. and *I. victoriensis*, which are separated by geography; *I. victoriensis* is found only in eastern Australia (Victoria and Tasmania) and *I. woyliei* only in the south west region of Western Australia, a distance of approximately 3,500 km. This geographical separation reflects the current allopatric distribution of the primary hosts for these species: *Vombatus ursinus* (the common wombat) for *I. victoriensis* and *B. penicillata* for *I. woyliei*. Until recently, however, these hosts were quite possibly sympatric species. *Bettongia penicillata* was once the most common and widest ranging of all potoroids covering most of southern Australia, but by the 1970’s was extinct from all regions except the south-western corner of Australia [27]. Theoretically, prior to European settlement genetic exchangeability should have been possible but is not evident in these results, again providing support for species status.

Molecular characterisation has been used extensively both to confirm tick species and to further our understanding of the phylogenetic relationships within various genera [7, 18, 28–32]. To achieve this, commonly used genetic markers have included the 12S and 16S ribosomal DNA, nuclear ribosomal internal transcribed spacer 2, and the mitochondrial *cox*1 gene. A recent paper assessing the effectiveness of these genetic markers concluded the *cox*1 gene was the most successful for tick species [33] and certainly the present study found this gene to be successful in unambiguously distinguishing between Australian *Ixodes* species.

### Host-parasite ecology

Based on the long-term data collected, it appears this tick has a strong predilection for *B. penicillata*, with 42% of *Ixodes* infections identified as *I. woyliei* n. sp*.* The two exceptions included two *I. o. fusciventer* and one *M. lagotis*, which may represent the ability for this novel species to use alternate sympatric hosts, or perhaps these represent accidental hosts. The *I. o. fusciventer* observation was made during early sympatric trapping sessions at Karakamia Wildlife Sanctuary in 2006; however *I. woyliei* was not detected in subsequent trappings of *I. o. fusciventer,* within two indigenous *B. penicillata* populations (Dryandra Woodland and the Upper Warren Region). In addition, a recent study investigating parasitism in urban populations of *I. o. fusciventer* that were not sympatric with *B. penicillata* sampled 287 individuals and *I. woyliei* was not detected (Hillman, pers. com.). Less information is available regarding the *M. lagotis* finding, except that this animal was located in an animal rehabilitation centre that was also known to frequently house *B. penicillata*. Whether there was a chance of enclosure contamination between these two hosts is speculative, but remains a possibility. Although the sample size for *I. o. fusciventer* is low and not all sympatric marsupial species (e.g. kangaroos) were sampled, the results suggest that *B. penicillata* is the preferred host for this tick. This apparent host preference displayed by *I. woyliei* n. sp. may be explained by an ecological link between a nidiculous tick species and a nest dwelling host. *Bettongia penicillata* individuals utilise several nests, normally located under grass trees (*Xanthorrhea* spp.), throughout their home range [34]. Transmission could largely be confined to *B. penicillata* if ticks detach, undergo development, and relocate to another host within these refuge sites. Depending on the frequency of nest sharing between alternate host species, of which *I. o. fusciventer* is most ecologically similar [35], this tick may simply be influenced by host specificity to these nests. The nidiculous nature of the new tick species may also explain the absence of male specimens detected from hosts, if mating occurs within nests with minimal time spent on the host.

The vectorial capacity of this novel tick species is unknown. Of particular concern for *B. penicillata* is the transmission of trypanosomes (protozoan blood parasites), which have been implicated in the recent population declines of this host [36, 37]. However, the *Trypanosoma* species (*T. copemani* and *T. vergrandis*) detected in *B. penicillata* have also been detected in other marsupials, suggesting that a generalist vector is responsible [38–42]. *Ixodes woyliei* n. sp. would not be considered a generalist tick and therefore less likely to be the vector for these blood parasites.

When considering the critically endangered status of *B. penicillata*, having undergone a 90% decline in seven years [13], and the apparent host specificity of *I. woyliei*, there is a very real risk of a future co-extinction event. Despite the recent dramatic decline in *B. penicillata* numbers, the data presented here suggest *I. woyliei* is maintaining a strong connection to *B. penicillata.* Co-extinction is of increasing importance as we discover more about wildlife host-parasite relationships and the possible flow-on effects these events can cause [43, 44]. Also of consideration for this tick species is the risk of extinction through translocation events. *Bettongia penicillata* is currently the focus of intense conservation management strategies involving the frequent and wide scale translocation of this species across Australia [45]. Some translocation protocols involve deliberate treatment for parasites (commonly with Ivermectin; [16]) with ticks often eliminated at the point of translocation [46].

If hosts are not treated, the ability of the tick population to establish in a new host population can be reliant on the number of ticks and hosts translocated [47], and suitability of the new ecological habitat for survival during off-host development phases. Within this study the Karakamia Wildlife Sanctuary site consists of a translocated *B. penicillata* population that also hosts *I. woyliei*, suggesting these ticks can survive translocation under the right conditions. Whether this tick is able to adapt to a wider geographical region (outside south-western Australia) or is restricted to a specific ecological biome is unknown but in some cases ticks have been found to have a narrower range in habitat than their host [48]. More research is required to understand how the ecology of *I. woyliei* n. sp. is influencing this strong host association, and what importance this new tick has for its critically endangered marsupial host.

## Conclusions

Morphological and molecular data have confirmed the first new Australian *Ixodes* tick species described in over 50 years, *Ixodes woyliei* n. sp. which has a high predilection for the critically endangered marsupial *B. penicillata*. The implications for this host-parasite relationship are unclear but there may be potential for a future co-extinction event. In addition, new molecular data have been generated for *I. australiensis*, *I. tasmani* and *I. victoriensis* and for the first time molecular support has been provided for the subgenus *Endopalpiger*, as initially described by Roberts [12]. These genetic data may also provide essential information for future studies relying on genotyping for species identification or for those tackling the phylogenetic relationships of Australian *Ixodes* species.

## Abbreviations

ANIC: Australian National Insect Collection
*cox*1: cytochrome *c* oxidase subunit 1 gene
WAM: Western Australian Museum
